# Health Effects of Alternate Day Fasting Versus Pair-Fed Caloric Restriction in Diet-Induced Obese C57Bl/6J Male Mice

**DOI:** 10.3389/fphys.2021.641532

**Published:** 2021-02-26

**Authors:** Chloe G. Henderson, Damian L. Turner, Steven J. Swoap

**Affiliations:** Department of Biology, Williams College, Williamstown, MA, United States

**Keywords:** ADF, CR, high-fat diet, intermittent fast, obesity, glucose

## Abstract

Alternate day fasting (ADF) induces weight loss and improves various markers of health in rodents and humans. However, it is unclear whether the benefits of ADF are derived from the lower caloric intake of ADF or from the 24-h fasting period. Therefore, this study directly compared selected markers for health – such as glucose control, body weight, liver triglycerides, T cell frequencies, and others – in high-fat (60% calories from fat) diet-induced obese mice subjected to either ADF or caloric restriction (CR). Obese mice were randomly assigned to one of four groups: (1) ADF: remained on the high-fat diet, but fed on alternate days (*n* = 5), (2) PF: remained on the high-fat diet, but pair-fed to the ADF group (*n* = 5), (3) LF: moved to a chow *ad libitum* diet (*n* = 5; 17% calories from fat), and (4) HF: remained on the high-fat *ad libitum* diet (*n* = 5). An additional group of non-obese mice maintained on a chow diet since weaning were used as controls (CON: *n* = 5). After 10 weeks, ADF, PF, and LF mice ate fewer kcals, had a lower body mass, had smaller epididymal fat pads, improved glucose tolerance, and had a lower hepatic triglyceride content relative to HF mice (*p* < 0.05), but none reached that of CON mice in these measures. T cell frequencies of the spleen, blood, and mesenteric lymph nodes were reduced in ADF, PF, and HF compared to the CON group. Importantly, there were no significant differences between the ADF and PF groups in any of the measurements made in the current study. These data suggest that ADF, PF, and LF diets each lead to improved markers of health relative to high-fat diet-induced obese mice, and that the caloric restriction associated with ADF is the major factor for the noted improvements.

## Introduction

Intermittent fasting can induce weight loss, improve glucose control, and improve lipid profiles among other physiological improvements in both humans and rodents (Varady and Hellerstein, 2007; Bhutani et al., 2013; Varady et al., 2013; Catenacci et al., 2016; Dedual et al., 2019). Alternate day fasting (ADF) is a form of intermittent fasting defined by alternating days of fasting (with full access to water and other calorie-free beverages) and *ad libitum* eating. ADF is an effective form of intermittent fasting that leads to weight loss, specifically fat mass (Varady et al., 2011; Baumeier et al., 2015; Catenacci et al., 2016; Joslin et al., 2017; Trepanowski et al., 2017; Gabel et al., 2019; Parvaresh et al., 2019), improved glucose control and insulin sensitivity (Halberg et al., 2005; Heilbronn et al., 2005; Yang et al., 2016; Swoap et al., 2019), and lowered circulating lipids and cholesterol in both rodents and humans (Mahoney et al., 2006; Bhutani et al., 2013; Varady et al., 2015). In rodent models, ADF has also been shown to increase cancer survival (Descamps et al., 2005; Varady et al., 2009; Xie et al., 2017), increase mean and maximal life span (Anson et al., 2003; Descamps et al., 2005), and reduce the effects of specific diseases such as asthma (Johnson et al., 2007). Importantly, ADF is a form of caloric restriction (CR) in that over 2 days, an individual will consume less than they would by eating *ad libitum* for 2 days (Anson et al., 2003; Caro et al., 2008; Catenacci et al., 2016; Joslin et al., 2017; Trepanowski et al., 2017). Because the ADF is a form of CR, a pair-fed group is also a form of CR.

Continuous CR has long been known to have similar beneficial metabolic and health effects as ADF (Spindler, 2010; Speakman and Mitchell, 2011). One question that remains open is whether the health improvements from ADF derive from the decreased caloric intake over a 48-h period, or from the extended fasting period on the fasting day. One mouse study showed lower fasting glucose and insulin in ADF mice as compared to both control and pair-fed mice (Anson et al., 2003). Human studies that directly compare ADF with CR have found similar weight loss effects (Varady et al., 2011; Catenacci et al., 2016; Trepanowski et al., 2017; Gabel et al., 2019). Some such studies show ADF as superior to daily CR for health benefits that include lowered systolic blood pressure and fasting plasma glucose (Gabel et al., 2019; Parvaresh et al., 2019). Other measures have been shown to not differ between ADF and CR in adults; these include body composition (in terms of total fat and lean muscle mass), serum cholesterol, HDL, LDL, triglycerides, insulin sensitivity, blood pressure, heart rate, fasting insulin, fasting glucose, C-reactive protein, and homocysteine (Catenacci et al., 2016; Trepanowski et al., 2017; Gabel et al., 2019). Therefore, the differential health benefits of CR, by means of pair-feeding, compared to ADF remain unclear.

While the mouse is only a model for human responses to diet manipulation, the mouse model allows for precise pair-feeding to examine whether the benefits of ADF are a result of the extended fast or from the reduction in caloric intake. We have previously shown that obese high-fat fed male C57Bl/6J mice have significant health gains (in terms of body weight (BW) and glucose regulation) when they undergo ADF while continuing on the high-fat diet (Joslin et al., 2017). Given the prevalent association of glucose dysregulation with obesity, many measures of glucose regulation [e.g., fasting blood levels, glucose tolerance tests, insulin-assisted glucose tolerance tests (IAGTs)] are used to assess the efficacy of ADF and CR (Varady et al., 2010; Baumeier et al., 2015; Hatting et al., 2017; Smith et al., 2019; Swoap et al., 2019). Plasma corticosterone and IL6, markers of stress and inflammation, respectively, have been shown to increase with a high-fat diet (Emerson et al., 2017; Appiakannan et al., 2019). Additionally, IL6 has been shown to play a role in insulin resistance in both animals and humans while also possibly contributing to fatty liver disease when present in excess (Vozarova et al., 2001; Senn et al., 2002; Emerson et al., 2017). Diet-induced obesity has been associated with impaired immune responses and reduced numbers of memory T cells in response to infection (Smith et al., 2007; Karlsson et al., 2010). Therefore, we sought to determine whether there were systemic changes in the frequencies of T cell populations in circulation and within lymphoid organs as a result of the various diets. Overall, we employed a diverse panel of these health markers as above to examine the potential differences between the dieting strategies of ADF and pair-fed CR.

## Materials and Methods

### Animals and Animal Diets

Thirteen-week-old diet-induced obese C57BL/6J male mice were delivered to Williams College from Jackson Laboratory (Stock No: 380050; Bar Harbor, Maine). At 6 weeks of age, these mice had been placed on a high-fat diet to induce obesity (60% of calories from fat, 20% from carbohydrates, and 20% from protein with a caloric density of 5.2 kcal/g, Research Diets D12492). Upon arrival at Williams College, the mice remained on the high-fat diet and were given 5 days to acclimate to the vivarium before the onset of the experimental diets. Thereafter, the mice were weight-matched and allocated into four diet regimens (*n* = 5 per group): (1) high-fat diet ADF. ADF treatment was defined by 24 h of *ad libitum* feeding followed by 24 h of fasting. The amount of food provided at the dark phase was weighed, as was the amount of food removed at the onset of the dark phase on the next day to calculate food consumption. (2) High-fat pair-fed (PF). The food consumed from all members of the ADF group was averaged from their fed day. The PF group then received half of this amount on each of the next 2 days, such that they were pair-fed over a 48-h period. The PF group was always 2 days behind the other experimental groups. The PF group was provided food at the onset of the dark phase on each day. (3) Chow food *ad libitum* (LF). The chow food, considered a low-fat food, contained 17% of calories from fat sources, 58% from carbohydrates, and 25% from protein with a caloric density of 3.1 kcal/g (Envigo Teklad LM-485). (4) High-fat food *ad libitum* (HF). The mice in this group were maintained on the high-fat food throughout the duration of the experiment. Concurrently, a group (CON: *n* = 5) of 14-week-old male C57BL/6J mice were raised at Williams College after two breedings from parents purchased from Jackson Labs, and had *ad libitum* access to the low-fat chow diet before and throughout the study as a control (Envigo Teklad LM-485). Food intake and BW were measured daily in the hour before the onset of the dark phase. All mice were singly housed in clear, standard, polycarbonate mouse cages with metal wire tops, maple sani-chip^®^ bedding (Envigo Teklad 7090), shredded paper (EnviroDri) for nesting, and *ad libitum* access to water. Cages were sustained in a 12:12 h light:dark phase at 23°C. Only male mice were used for two reasons: (1) because their metabolic response to a HF diet is substantially more pronounced than the female response to a HF diet, and (2) the epididymal fat pad is easily isolated from males. All experimental treatment was approved by the Williams College Institutional Animal Care and Use Committee and complied with the Guide for the Care and Use of Laboratory Animals (protocol # SS-N-17).

### Glucose Tolerance Test and Insulin-Assisted Glucose Tolerance Test

A Glucose Tolerance Test (GTT) was performed on each mouse from all diet regimens at 20 weeks of age, 6 weeks into the feeding paradigm, as described by Schindler et al. (2014). At the onset of the light phase, 6 h before testing, mice were weighed with continued *ad libitum* access to water, and food was removed. After the 6-h fast, each mouse had its tail nipped with a disposable sterile surgical blade. The drop of blood from the tail was used to measure fasting blood glucose concentration (*t* = 0) with a glucometer (Nova Max Plus). Immediately after, the mouse was intraperitoneally injected with 1 g glucose/kg of BW in 0.1–0.2 cc of sterile saline. Blood glucose concentrations were measured at 20, 40, 60, 90, and 120 min after injection. An IAGT was performed on each mouse from each diet group at 23 weeks of age, 9 weeks into the feeding paradigm, as previously described (Reynolds et al., 2009; Joslin et al., 2017). The same 6 h fasting protocol from the GTT was followed, except that the mice were intraperitoneally injected with a cocktail of glucose (1 g/kg BW) and insulin (0.75 U/kg BW; Sigma Aldrich I0908) in 0.1–0.2 cc of sterile saline. Thereafter, blood glucose concentration measurements were taken at *t* = 20, 40, 60, 90, and 120 min after injection. The area under the curve measurement (AUC) was calculated in Excel by adding two consecutive data points, dividing by two, and multiplying by the difference in time per the formula [(B1 + B2)/2) × (A2−A1)] where column A contains the time in minutes and column B contains the blood glucose measurement at that time. This formula was applied to the full duration of the blood glucose test and values were summed to calculate the total AUC.

### Blood and Tissue Collection

Mice were euthanized while under 4% isoflurane anesthesia after 10 weeks of their specified diet treatment. ADF mice were euthanized after a fed day. All mice were fasted for 6 h before euthanasia. Blood was withdrawn from the left ventricle of the heart into a syringe pretreated with EDTA, transferred to a 1.7-ml microcentrifuge tube, and centrifuged at 10,000 × *g* for 2 min. Plasma was aliquoted into several tubes, flash-frozen in liquid nitrogen, and stored at –80°C. The cellular component of the blood was treated as below. Liver lobes and epididymal fat pads were excised, flash frozen in liquid nitrogen, and stored at –80°C. Epididymal fat pads were also weighed immediately after excision. The spleen and mesenteric lymph nodes were dissected and processed immediately as described below.

### Preparation of Cell Suspensions and Flow Cytometric Analysis

Spleens were mechanically disrupted using the cap of an Eppendorf tube to grind tissue against a piece of 100 μm nylon mesh that was placed in a petri dish. The disrupted spleen tissue was then incubated in red blood cell lysing buffer (Biolegend, San Diego, CA, United States) for 2 min and then resuspended in RPMI media. Mesenteric lymph nodes were mechanically disrupted in RPMI media alone. Cell pellets from blood samples were resuspended in red blood cell lysing buffer (Biolegend, San Diego, CA, United States), incubated for 2 min, centrifuged, then incubated in lysis buffer a second time. Cell suspensions from all tissues were filtered through 100 μm nylon mesh and aliquots containing 1 × 10^6^ – 5 × 10^6^ cells stained with the fluorescently conjugated antibodies: CD4 (Pacific. Blue), CD8 (PE), and CD3 (BV785) (Biolegend, San Diego, CA, United States). Samples were analyzed using a Beckman Cytoflex flow cytometer and analyzed using Flowjo flow cytometry analysis software (FlowJo LLC, OR).

### Blood Analytes

Plasma corticosterone concentrations were measured using the Mouse Corticosterone ELISA Kit (Arbor Assays^®^, Ann Arbor, Michigan) according to the manufacturer’s instructions. Plasma and liver homogenate triglyceride concentrations were measured using the Triglyceride Colorimetric Assay Kit (Cayman Chemical, Ann Arbor, Michigan) according to the manufacturer’s instructions. Frozen livers were homogenized in NP40 Substitute Assay Reagent from the Triglyceride Colorimetric Assay Kit, centrifuged at 10,000 × *g* for 10 min, and triglycerides were measured in the supernatant according to the manufacturer’s instructions. Plasma interleukin 6 (IL6) concentrations were determined using a Mouse IL-6 ELISA Kit (RayBio^®^, Norcross, Georgia) following the manufacturer’s instructions.

### Statistical Data Analysis

All results are expressed as means ± standard deviation. Statistical analyses were performed in SPSS 25.0 to conduct 1 × 5 ANOVAs, followed by LSD *post hoc* testing on all variables except kcal consumed. For this test, we used a 1 × 4 ANOVA and left out the PF group as the food given to this group was not a measured variable. A non-paired student *T*-test was used to analyze initial BWs. Only *p*-values < 0.05 were reported as statistically significant.

## Results

### Body Weight and Food Intake

**FIGURE 1:**
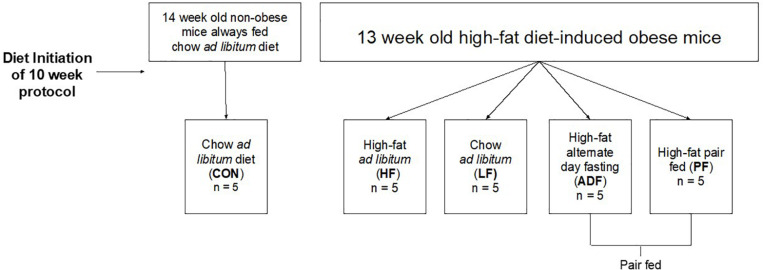
Schematic of experimental grouping.

**FIGURE 2:**
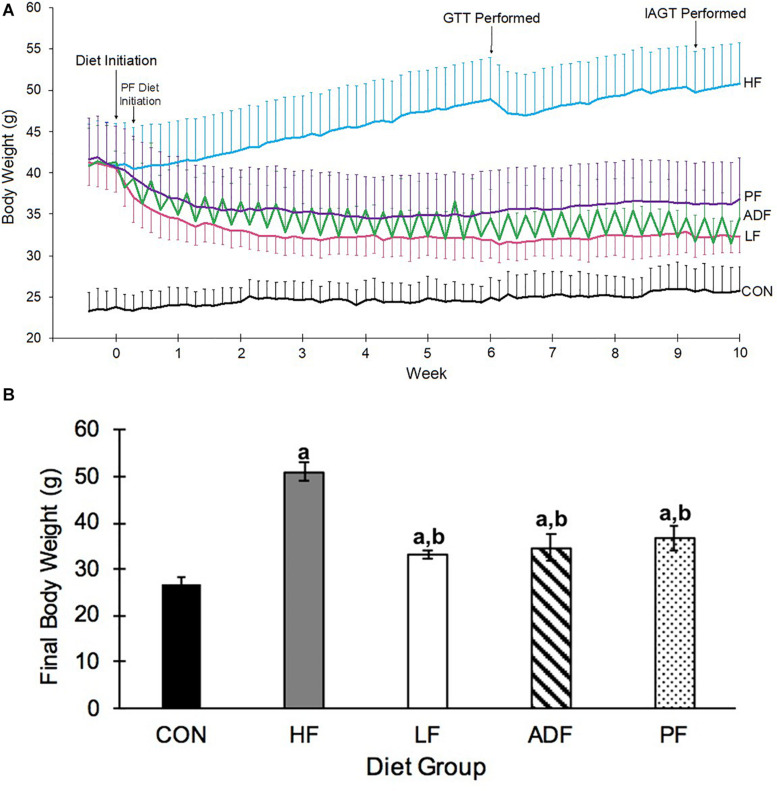
Changes in body weight. **(A)** Body weight over 10 weeks of designated diet. Mice on the high-fat diet leading up to the dietary manipulation weighed 41.0 ± 3.3 g. The alternate-day-fast on a high-fat diet (ADF: green) and the switch to *ad libitum* chow diet (LF: pink) commenced at Day 0, whereas the pair fed group (PF: purple) that received the same amount of high-fat food as the ADF, began on Day 2. The other two groups of mice were those that remained *ad libitum* on the high-fat diet (HF: blue), and the mice that were maintained on an *ad libitum* chow diet (CON: black). The daily variation in BW of the ADF group was due to alternating days of feeding and fasting. Mice were weighed daily in the hour before the onset of the dark phase. **(B)** Body Weight on last experimental day. Alternate day fasting, caloric restriction, and switching to a chow *ad libitum* diet all led to a significantly lower BW as compared to the BW of HF *ad lib* diet-induced obese mice. Body weights shown are after a 6 h fast and after a fed day for ADF mice on the last experimental day. a: *p* < 0.05 vs. CON; *b*: *p* < 0.05 vs. HF.

At the onset of the dietary manipulations (see Figure 1 for a schematic), the mice consuming the high-fat diet weighed 41.0 ± 3.3 g, significantly more than those consuming the chow diet, 24.0 ± 2.5 g (*p* < 0.05). After initiation of the dietary manipulation on the high-fat fed mice, BW rapidly decreased for the three experimental diet regimens (ADF, PF, and LF) (Figure 2A). Roughly 2 to 3 weeks into the dietary manipulation, BWs for all of the groups stabilized and were maintained throughout the remainder of the study, except for BW of the HF group which continued to increase throughout all 10 weeks (Figure 2A). At the end of 10 weeks, the BW of CON and HF groups were statistically significant from all of the experimental diet regimens and from each other (*p* < 0.05) (Figure 2B). BW differences among the experimental diet regimens at the end of the 10 weeks were not significant from each other (Figure 2B).

**FIGURE 3:**
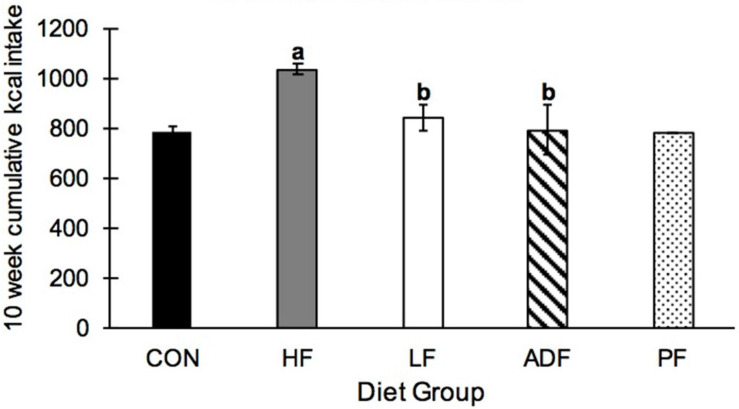
Cumulative kcal intake over 10 weeks. Mice in the HF group consumed significantly more kcals than all other diet regimens, with no differences among any of the other groups. a: *p* < 0.05 vs. CON; b: *p* < 0.05 vs. HF.

Over the 10-week experimental period, HF mice ate significantly more kcals than all of the other groups (Figure 3). CON, ADF, and LF did not consume significantly different amounts of kcals from each other (Figure 3). By definition, the PF group consumed the identical caloric intake as the ADF group, and therefore was excluded from the statistical analysis here. It is important to note that the experimental mice that were initially obese from the high-fat diet all continued to weigh more than CON mice (Figure 2) despite eating the same number of calories over the 10-week period (Figure 3).

### Blood Glucose, Glucose Tolerance Test, and Insulin-Assisted Glucose Tolerance Test

**FIGURE 4:**
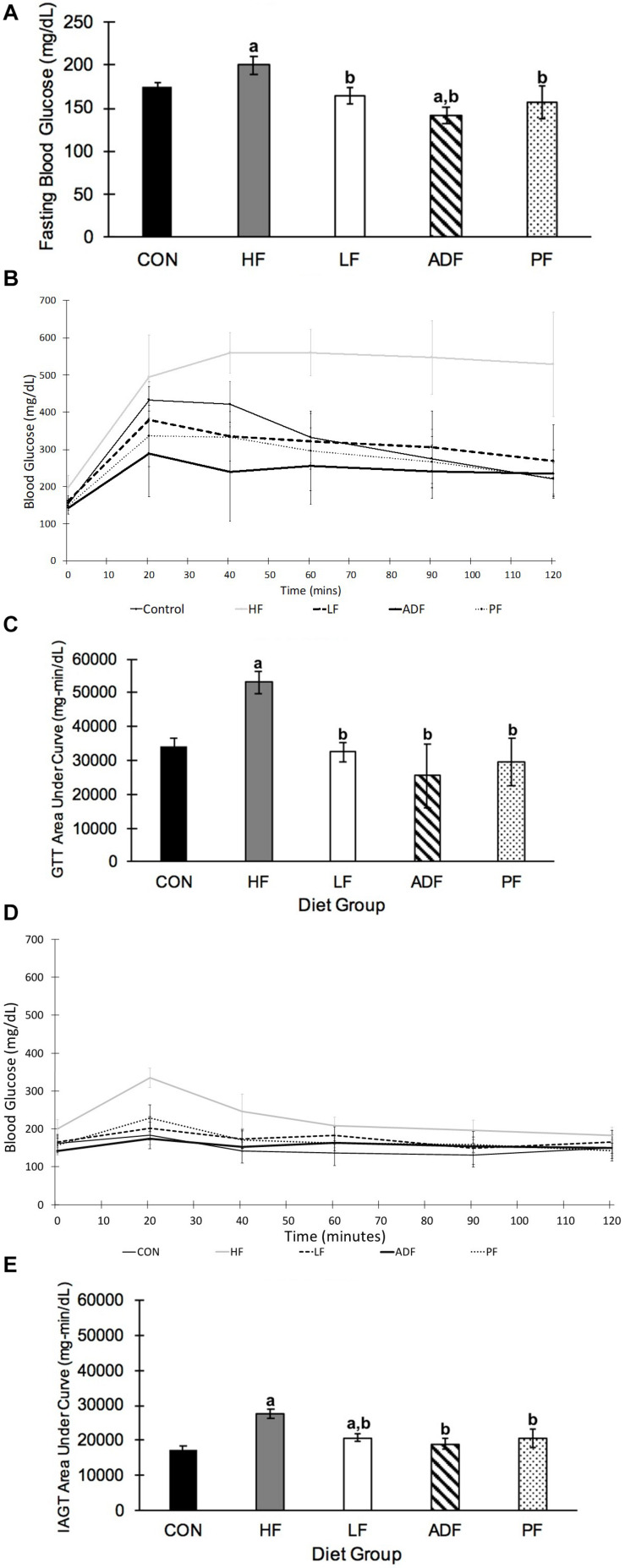
Blood glucose and glucose tolerance tests. **(A)** Fasting blood glucose. Blood glucose was measured after a 6 h fast, which started at the onset of the light phase. Blood glucose of ADF mice was measured after a fed day. Mice in the HF group had significantly higher fasting blood glucose than all other diet regimens (*p* < 0.05). ADF mice had significantly lower blood glucose compared to CON mice **(B)** Glucose tolerance test. A GTT was conducted after a 6 h fast, which began at the onset of the light phase. ADF mice were tested after a fed day. Samples were collected 20, 40, 60, 90, and 120 min after intraperitoneal injection of glucose. **(C)** Glucose tolerance test area under the curve (AUC). The “area under the curve” calculation was measured using the lowest blood glucose concentration from the GTT as a baseline. All mice were significantly more glucose tolerant than HF mice. **(D)** Insulin assisted glucose tolerance test. An IAGT was conducted after a 6 h fast, which began at the onset of the light phase. ADF mice were tested after a fed day. Mice were 23 weeks of age and 9 weeks into their experimental diets at the time of the IAGT. Mice were given intraperitoneal injections of a cocktail of 1 g glucose/kg body weight with 0.75 U/kg body weight in 0.1–0.2 cc of sterile saline. **(E)** Insulin assisted glucose tolerance test area under the curve (AUC). HF mice had a significantly larger AUC than all other diet regimens, once again suggesting that they are more glucose intolerant than the other diet regimens. a: *p* < 0.05 vs. CON; b: *p* < 0.05 vs. HF.

After 6 weeks of the dietary treatment, fasting blood glucose was significantly lower in all of the groups as compared to the HF mice (Figure 4A). ADF mice had a significantly lower blood glucose from CON mice, but no different to any of the other groups (Figure 4A). In the glucose tolerance test (GTT), the HF mice were the least glucose tolerant with the greatest area-under-the-curve (AUC) measurement, statistically higher than each of the other groups (Figures 4B,C). There were no differences among the ADF, PF, LF, and CON groups for the GTT AUC (Figure 4C). However, the peak blood glucose value during the GTT for the ADF group (289 ± 117 mg/dl) was significantly lower than that of the CON (439 ± 50 mg/dl), HF (574 ± 42 mg/dl), and LF (400 ± 97 mg/dl) groups. Meanwhile, the peak blood glucose value during the GTT for the PF group (361 ± 122 mg/dl) was only significantly lower than that of the HF group.

For the IAGT administered 9 weeks into the dietary regimen (Figures 4D,E), the AUC value of the HF mice was significantly greater than all other groups. As with the GTT AUC, there were no differences among the other four groups. It is important to note that despite the rapid rise in blood glucose in HF mice during the IAGT, circulating blood glucose steadily decreased, as opposed to the GTT. The LF response was significantly greater than that of the CON mice (Figure 4E).

### Epididymal Fat Pads Mass and Liver Triglycerides

**FIGURE 5:**
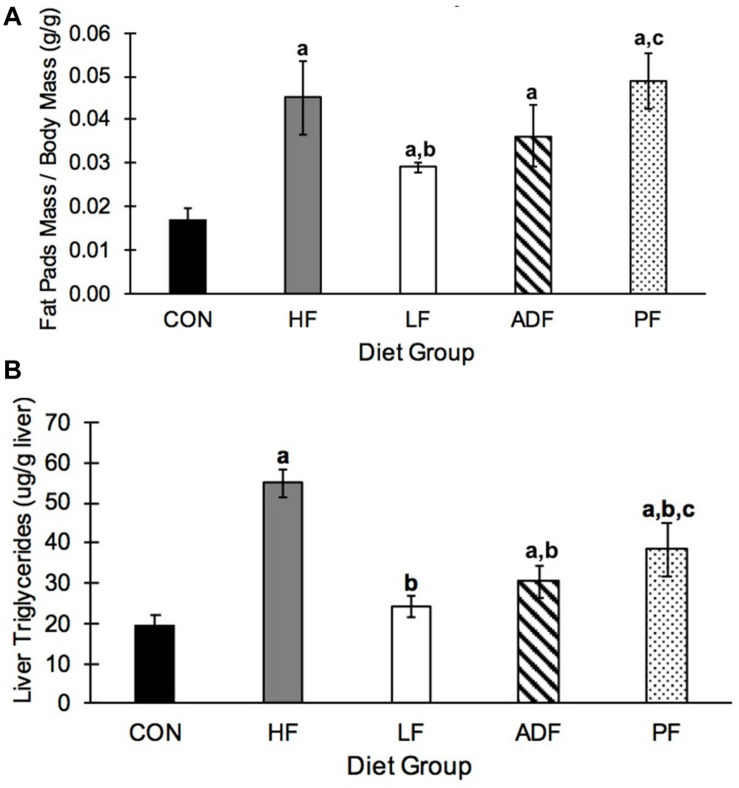
Epididymal fat and hepatic triglycerides. **(A)** Average mass of both epididymal fat pads relative to body weight. CON mice had significantly smaller epididymal fat pads relative to BW compared to all other groups. In addition, the relative size of epididymal fat pads in the LF group were significantly smaller than those from HF and PF mice. **(B)** Liver triglyceride concentrations. HF liver triglyceride concentrations were triple that seen in CON mice. All diet regimens reduced this measure, with moving to a LF diet as the most effective. a: *p* < 0.05 vs. CON; b: *p* < 0.05 vs. HF; c: *p* < 0.05 vs. LF.

Epididymal fat pads mass varied widely among the groups (Figure 5A). HF (2.24 ± 0.73 g) and PF (1.81 ± 0.31 g) mice had significantly heavier epididymal fat pads than all other groups (CON: 0.48 ± 0.19 g; LF: 0.96 ± 0.14 g; ADF: 1.25 ± 0.22 g), but not from each other. When epididymal fat pad masses were normalized to control for BW, CON mice had a lower relative fat pads mass than all of the other groups (Figure 5A). Only mice in the LF group had smaller relative fat pad mass as compared to the HF group (*p* = 0.044). The relative fat pad mass from PF mice was not significantly different from that of the ADF mice (*p* = 0.080).

Liver triglycerides were measured in liver homogenates. HF mice had significantly more hepatic triglycerides than all other diet regimens (Figure 5B). The two groups of mice consuming chow food, the CON group and the LF *ad libitum* group, had lower liver triglycerides than all of the groups on the high-fat food, except ADF. The liver triglyceride content was not different between the PF group and the ADF group (Figure 5B).

### T Cell Frequency in the Spleen, Blood, and Mesenteric Lymph Nodes

**FIGURE 6:**
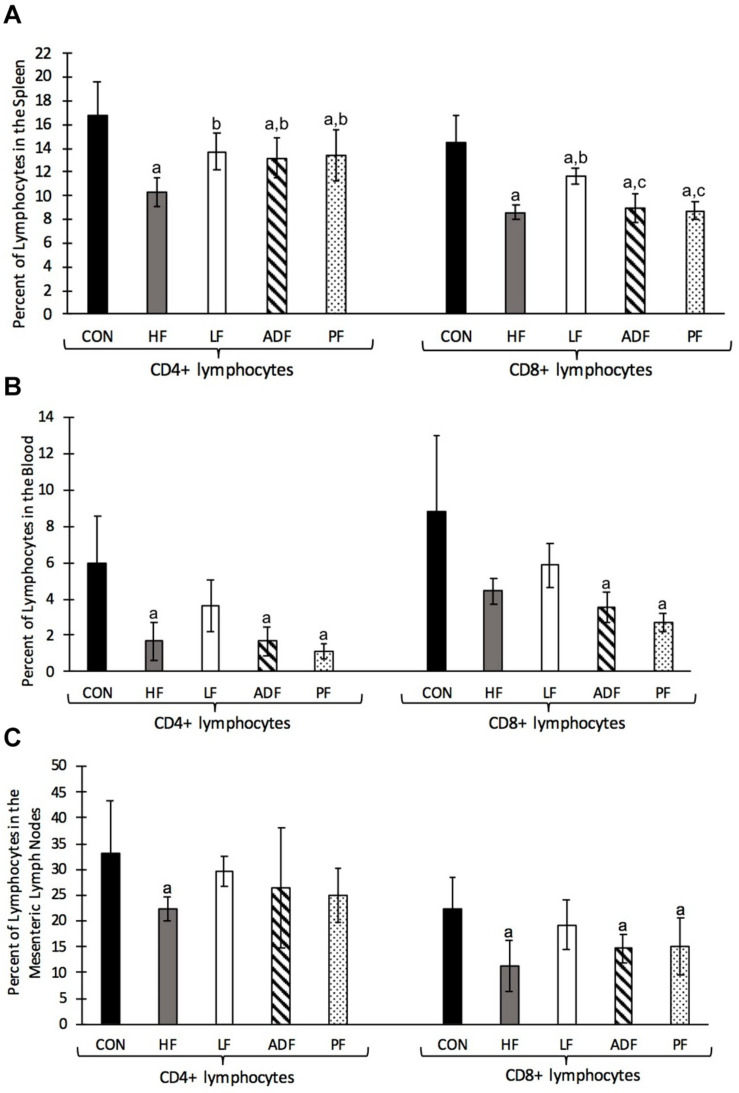
Single cell suspensions from the spleen, blood, and mesenteric lymph nodes were stained with anti-CD3, anti-CD4, and anti-CD8 monoclonal antibodies and analyzed by flow cytometry. The lymphocyte population was gated based on FSC vs SSC profiles then CD3 + cells gated and used as the parent population for CD4 + and CD8 + T cell identification. Graphs show mean and SD of the percentage of CD4 + and CD8 + T cells of lymphocytes. **(A)** Frequency of T cells in the spleen. All diet regimens had a lower percentage of splenic CD4 + and CD8 + T cells compared to the CON group. The LF group had a significantly higher percentage of CD4 + and CD8 + T cells compared to the HF group. **(B)** Frequency of T cells in the blood. HF, ADF, and PF groups had reduced frequencies of CD4+ and CD8+ T cells in the blood, while the LF group showed no significant reduction. **(C)** Frequency of T cells in the mesenteric lymph node. HF, ADF, and PF groups had reduced frequencies of T cells in the mesenteric lymph nodes while the LF group showed no significant reductions in T cell frequency. a: *p* < 0.05 vs. CON; b: *p* < 0.05 vs. HF; c: *p* < 0.05 vs. LF for each respective lymphocyte population.

Single cell suspensions derived from spleen, blood, and mesenteric lymph nodes were stained with T cell specific antibodies and analyzed by flow cytometry. T cell frequencies in the spleen, blood, and mesenteric lymph nodes were reduced in all diet regimens compared to the CON group (*p* < 0.05) (Figure 6). The lower splenic T cell frequencies were due to reductions in the frequencies of both major subsets of T cells (CD4+ and CD8+) (Figure 6A). Interestingly, the reduction in splenic CD4 + T cell frequency was less in the LF, ADF, and PF groups than in HF mice (*p* < 0.05) (Figure 6A). Splenic CD8 + T cells were also less dramatically reduced in the LF mice compared to the HF group (*p* < 0.05) (Figure 6A). There were no significant reductions in blood CD4+ and CD8+ T cell frequencies in the LF group compared to the HF group (Figure 6B); however, both CD4+ and CD8+ T cell frequencies were reduced in ADF and PF mice (*p* < 0.05) (Figure 6B). Likewise, there were no significant reductions in mesenteric lymph node CD4+ and CD8+ T cell frequencies in the LF group compared to CON (Figure 6C). Mesenteric lymph node CD8+ T cell frequencies in the ADF and PF groups were reduced compared to CON (*p* < 0.05) (Figure 6C) but CD4+ T cell frequencies were not significantly different (Figure 6C).

### Plasma Measures

Plasma corticosterone, plasma IL6, and plasma triglycerides were not significantly different among all of the groups (Table 1).

**TABLE 1:** Experimental values of plasma corticosterone, IL6, and triglycerides.

**Group**	**Plasma corticosterone (ng/mL)**	**Plasma IL6 (pg/mL)**	**Plasma triglyceride (mg/dL)**
CON	49 ± 12	33 ± 10	42 ± 9
HF	128 ± 40	31 ± 5	50 ± 11
LF	52 ± 14	29 ± 8	57 ± 11
ADF	61 ± 14	31 ± 9	63 ± 3
PF	92 ± 25	26 ± 4	41 ± 4

## Discussion

In the current experiment, we wanted to examine whether the improvement in select health markers seen in high-fat diet-induced obese mice from ADF (Joslin et al., 2017) was due to the CR effect of ADF or due to the fasting nature of the ADF regimen. We first confirmed that ADF in this model leads to lower caloric intake (Figure 3). Indeed, the ADF protocol resulted in ∼20% reduction in food intake, a little less than what we have measured previously at ∼30% (Joslin et al., 2017), but approximately what others have found (Caro et al., 2008; Gabel et al., 2019). By definition, the PF group received the same amount of high-fat food as the ADF group. We note that this level of caloric intake for the ADF group (and PF group) was not different from either group on the *ad libitum* chow diets (CON and LF: Figure 3), as has been seen previously (Gotthardt et al., 2016). That is, all of the dietary manipulations here resulted in similar caloric intake. We found significant improvement among all of those mice that ate fewer calories than the HF *ad libitum* group, but few differences among the diet groups themselves. For mice, it appears that the most important variable for improved health markers measured here from an obese state is the reduction of calorie intake, and not the length of fasting time. In other words, reducing caloric intake through either changing the composition of the diet (i.e., the LF group), eating every other day (ADF group), or eating less every day (PF group), results in similar health outcomes. Each of these outcomes is outlined below, starting with BW.

Body weight dropped precipitously in all of the dieting groups, particularly during the first 1–2 weeks of the feeding paradigm, but did not reach the BW of CON mice. For the experimental diets, BW did not continue to decrease after the initial 2 weeks, suggesting that there may be a limit to the BW loss induced by diet alone in these feeding regimens. Others (Baumeier et al., 2015; Gotthardt et al., 2016; Joslin et al., 2017; Kusuoka et al., 2018) have shown that some version of ADF, CR, or returning to a LF diet causes weight loss in a diet-induced obese rodent model. We extend those findings here by directly comparing these dieting groups. We found that each of the diets is equally efficacious for BW control in previously obese mice.

Associated with the BW loss was an improvement in glucoregulatory control, which is important as a health marker due to the association of obesity with glucose dysregulation. We examined four related markers associated with glucose regulation including fasting blood glucose, peak blood glucose, response to a GTT, and response to an IAGT. Fasting blood glucose of HF mice was high (200 ± 25 mg/dL) suggesting that the high-fat diet-induced obese mice had blood glucose dysregulation. All diet groups showed a decreased fasting blood glucose relative to HF mice, as seen previously, with the ADF group exhibiting lower blood glucose despite the measurement taken after a fed day (Anson et al., 2003; Gotthardt et al., 2016; Yang et al., 2016; Liu et al., 2018). The improved glucose control seen in ADF mice compared to *ad libitum* controls is well known in rodents and humans (Joslin et al., 2017; Dedual et al., 2019; Swoap et al., 2019). We found that all of the diet manipulations lead to improved GTT responses relative to the HF group, confirming those findings. We extend those findings to show that there were no differences among the different dieting regimens, and in particular, the ADF group vs. the PF group (Figures 4B,C).

Another measure that can be associated with obesity is hepatic triglyceride content. We also observed a reduction in this health marker in the groups consuming fewer calories. Hepatic fat content is a critical contributor to non-alcoholic fatty liver disease (NAFLD) and studies have shown that ADF can be an effective diet therapy for NAFLD in both obese humans (Cai et al., 2019; Johari et al., 2019) and animals (Yang et al., 2016; Rusli et al., 2017). In our study, any form of CR significantly decreased hepatic triglycerides compared to HF mice (Figure 5B).

Switching to a chow diet from a high-fat diet (LF group) proved to be more effective than the CR associated with ADF and PF for two measures: epididymal fat pad mass and T cell frequency. Mice in the LF group showed significantly smaller fat pad mass on both an absolute level (see results) and on a body mass-specific level (Figure 5A) than the HF group, an observation not seen in either the ADF or PF groups. That is, epididymal fat pad mass was not dependent on BW. This suggests that the feeding regimen is the key factor in determining epididymal fat pad mass, not BW or caloric intake (Figure 5A). While we measured epididymal fat mass, and not whole-body fat mass, others have done so in mice and show significant decreases in body fat mass in mice undergoing the ADF protocol for both low-fat and high-fat food (Gotthardt et al., 2016; Smith et al., 2019). We used the epididymal fat pad as an easily dissected and self-contained tissue as one indicator of fat storage in the mouse. Finally, our results on T cell frequencies also suggest that the type of food consumed might be critical for immune cell homeostasis. Previous research has shown that chronic CR leads to a reduction in circulating monocytes (CD14+ and CD16+) in both mice and humans (Jordan et al., 2019), with enhanced T cell accumulation in the bone marrow (Collins et al., 2019). We observed that T cell frequencies were reduced in the spleen, blood, and mesenteric lymph nodes in all three HF groups (HF, ADF, and PF) (Figure 6). In all three tissues, the LF group showed smaller reductions in T cell frequencies (if any). These results suggest that diet regimens with high fat content might have a deleterious effect on T cell expansion, survival, or homeostasis. An alternate explanation for reduced T cell frequencies, however, might be the expansion of B cells, the other major lymphocyte population. It will be important to determine whether HF diet regimens reduce T cell numbers or increase B cell numbers in lymphoid organs and in circulation.

It is important to note that all measurements were made after a fed day for the ADF group. We have previously shown that there are differences in BW and glucoregulatory control after a fed versus a fasted day for obese mice on HF ADF (Joslin et al., 2017). Because measurements are known to be different between the fed day versus the fasted day, we wanted to test for changes after a day of food overconsumption, when a mouse might be metabolically worse than after the fasted day. We did not specifically note when mice in the ADF group ate or when the PF mice finished consuming their food. The specific timing of eating in these groups may be of interest for future studies.

The sample size in the current study was small (*n* = 5 per group), and we only studied male mice. It remains to be determined whether a similar phenotype will be found in female mice. Further, while the chow diet and HF diet used here is well defined for macronutrients (calories from carbohydrate, fat, and protein), the chow diet used is not well-defined for its micronutrient availability. Of note, the CON mice used in this study were derived from mice that were ordered from Jackson labs 4 months prior to the start of the study. These mice were bred twice without backcrossing at Williams College; therefore, we would not expect any drastic genetic changes between this group of mice and the 13-week-old obese mice ordered from Jackson labs.

In addition to treating obesity, ADF is also commonly used to improve health in non-obese conditions. In non-obese adults, ADF has been found to decrease fat mass, improve the fat-to-lean ratio, improve cardiovascular parameters such as decreased heart rate and blood pressure, and lower LDL, among others (Varady et al., 2013; Stekovic et al., 2019). This warrants further research into how the health benefits of ADF may compare to CR in a non-obese model.

To sum, the results of this study suggest that for BW and glucoregulatory action, the primary factor for improvement seen in obese mice exposed to ADF appeared to be the reduction of calories, and not the fasting inherent to the ADF model or to the type of calories consumed (low-fat or high-fat). Hepatic triglyceride content demonstrated a beneficial effect for the ADF vs. the PF group, however, the interpretation of this finding is limited by sample size. For T cell frequency, the high-fat diet seems to play a more important role, although some changes were seen with decreased caloric intake, particularly in the blood and mesenteric lymph nodes. Although the sample size in the current study was not large, the changes in these markers relative to the HF group were robust. Importantly, we found that all of the dieting groups displayed improvement in most of the measured markers over the 10-week feeding paradigm. Understanding the differing physiological effects of ADF versus continuous CR on a high-fat diet can help us in evaluating the efficacy of common dieting regimens as treatments for diseases such as obesity, NAFLD, and diabetes.

## Data Availability Statement

The raw data supporting the conclusions of this article will be made available by the authors, without undue reservation.

## Ethics Statement

The animal study was reviewed and approved by the Williams College Animal Use Committee.

## Author Contributions

CH and SS designed the research. CH, DT, and SS conducted the research and analyzed the data. CH wrote the manuscript. DT and SS edited the manuscript. SS had primary responsibility for final content. All authors read and approved the final manuscript.

## Conflict of Interest

The authors declare that the research was conducted in the absence of any commercial or financial relationships that could be construed as a potential conflict of interest.

## Abbreviations

ADF: alternate day fasting
AUC: area under the curve
CR: caloric restriction
GTT: glucose tolerance test
HF: high fat
LF: low fat
IAGT: insulin assisted glucose tolerance test
NAFLD: non-alcoholic fatty liver disease.
